# Using logistic regression to improve the prognostic value of microarray gene expression data sets: application to early-stage squamous cell carcinoma of the lung and triple negative breast carcinoma

**DOI:** 10.1186/1755-8794-7-33

**Published:** 2014-06-10

**Authors:** David W Mount, Charles W Putnam, Sara M Centouri, Ann M Manziello, Ritu Pandey, Linda L Garland, Jesse D Martinez

**Affiliations:** 1 Bioinformatics Shared Service, Arizona Health Sciences Center, The University of Arizona, Tucson, Arizona 85735, USA; 2 Department of Surgery, Arizona Health Sciences Center, The University of Arizona, Tucson, Arizona 85735, USA; 3 Arizona Comprehensive Cancer Center, The University of Arizona, Tucson, Arizona 85735, USA; 4 Department of Medicine, Arizona Health Sciences Center, The University of Arizona, Tucson, Arizona 85735, USA; 5 Department of Cellular and Molecular Medicine, Arizona Health Sciences Center, The University of Arizona, Tucson, Arizona 85735, USA

## Abstract

**Background:**

Numerous microarray-based prognostic gene expression signatures of primary neoplasms have been published but often with little concurrence between studies, thus limiting their clinical utility. We describe a methodology using logistic regression, which circumvents limitations of conventional Kaplan Meier analysis. We applied this approach to a thrice-analyzed and published squamous cell carcinoma (SQCC) of the lung data set, with the objective of identifying gene expressions predictive of early death versus long survival in early-stage disease. A similar analysis was applied to a data set of triple negative breast carcinoma cases, which present similar clinical challenges.

**Methods:**

Important to our approach is the selection of homogenous patient groups for comparison. In the lung study, we selected two groups (including only stages I and II), equal in size, of earliest deaths and longest survivors. Genes varying at least four-fold were tested by logistic regression for accuracy of prediction (area under a ROC plot). The gene list was refined by applying two sliding-window analyses and by validations using a leave–one-out approach and model building with validation subsets. In the breast study, a similar logistic regression analysis was used after selecting appropriate cases for comparison.

**Results:**

A total of 8594 variable genes were tested for accuracy in predicting earliest deaths versus longest survivors in SQCC. After applying the two sliding window and the leave-one-out analyses, 24 prognostic genes were identified; most of them were B-cell related. When the same data set of stage I and II cases was analyzed using a conventional Kaplan Meier (KM) approach, we identified fewer immune-related genes among the most statistically significant hits; when stage III cases were included, most of the prognostic genes were missed. Interestingly, logistic regression analysis of the breast cancer data set identified many immune-related genes predictive of clinical outcome.

**Conclusions:**

Stratification of cases based on clinical data, careful selection of two groups for comparison, and the application of logistic regression analysis substantially improved predictive accuracy in comparison to conventional KM approaches. B cell-related genes dominated the list of prognostic genes in early stage SQCC of the lung and triple negative breast cancer.

## Background

When commercial microarrays encompassing most of the human genome transcripts became available, much attention was focused upon gene expression patterns of primary tumors as indicators of likely disease progression. The presumption was that evidence of dysregulation of certain genes within the excised primary tumor could be used to improve the prognostic discrimination of clinical and pathologic staging alone [1,2], by indicating the likelihood [3-6] that dissemination of the tumor had already occured [7,8]. Although this strategy has yielded limited success with certain malignancies, the hope that microarray analysis would provide prognostic data complementary to clinical staging has largely remained unfulfilled [9-16]. This difficulty becomes quite evident when gene lists from similar studies are compared and show little if any overlap. By way of example, to date 13 analyses of large expression data sets of squamous cell carcinoma of the lung (SQCC) cases have been published [11,17-28]. However, the deduced gene profiles have very few genes in common [19], even when the same data set was analyzed independently by three different groups [18,20,22]. Similarly, Roepman, *et al*. [19], compiled prognostic genes from eight analyses of NSCLC and found only five of 327 genes in common. Three of the consensus genes were from two independent reports of the same data set [29,30].

Although a number of factors, from tissue acquisition to compilation of clinical data, conspire to complicate the task of identifying prognostic gene expressions (reviewed in [31,32]), we focus here upon two vital considerations in the analysis of microarray data sets: optimal use of clinical data and rigorous, robust mathematical analysis. In this report, we describe the application of the well-established statistical approach, logistic regression, to the analysis of large gene expression data sets which include corresponding clinical data, such as survival or therapeutic response. Typically, an expression data set is analyzed by (1) identifying individual gene expression variations which demonstrate the largest excursions within the data set; (2) grouping the cases into quantiles based on sorted expression values of these genes; (3) comparing survival between quantiles, using Cox proportional hazard models to stratify clinical data and Kaplan Meier (KM) plots [33]; (4) applying statistical tests to deduce the success of the quantiles in predicting survival; and (5) compiling a predictive “signature” or “metagene” and, often, constructing a mathematical formula in which expression values of the signature genes are weighted to optimize its predictive success.

Our approach differs substantively from KM analysis, and consequently circumvents several limitations of the methodology just described [34]. First, two classes of patient cases - equal in size - are compared (in this report, “earliest deaths” and “longest survivors”) to assess the accuracy of gene expression predictors; this strategy avoids relying upon KM survival plots, which are often based upon incomplete or heavily right-censored clinical data [35]. Second, after isolating a subset of genes which are highly variable across the entire data set, and using the groups just described, logistic regression is employed to identify those genes offering statistically significant predictive value, as judged by the area under the curve (AUC) of a receiver operating characteristic (ROC) plot and statistical examination of the logistic regression model [36,37]. This initial list of prognostic genes is further refined by first enlarging the two groups and then executing two sliding window analyses of the larger groups of early deaths and longest survivors. The final list of independently prognostic genes is validated by assigning training and testing subsets using a leave-one-out [38] or similar approach.

Our approach evolved as we sought to identify genes prognostic of early death or long survival in patients with early-stage SQCC, using a large published data set and accompanying clinical information [18]. In this report we describe our analytic process using logistic regression; we ultimately identified 24 genes which have excellent prognostic discrimination. Application of a conventional KM approach to the same data, however, succeeded in identifying only a minority of the 24 genes found by logistic regression. Interestingly, immune cell-related genes, especially those associated with the B cell lineage, dominated the 24-gene list, in agreement with a substantial body of other experimental evidence, as recently reviewed by Whiteside [39]. As further proof of the utility of the logistic regression method for identifying prognostic genes, we extended the same computational methods to a triple negative breast carcinoma data set. Treatment of this disease presents similar clinical challenges to SQCC [40]. Remarkably, the analysis revealed a major role for B-cell and also for other immune-related genes in disease recurrence after tumor resection.

## Methods

All data analyses including statistical calculations, graphical displays, and probe annotations were produced using R programming tools (http://www.R-project.org) and BioConductor libraries (http://www.bioconductor.org). For the lung study, a previously published data set [18] of gene expression measurements of tissue samples of non-small-cell lung cancer on Affymetrix HGU133A microarrays was obtained from the GEO (gene expression omnibus data set) at NCBI (http://www.ncbi.nlm.nih.gov/gds). “The samples were collected from patients from the University of Michigan Hospital between October 1991 and July 2002 with patient consent and Institutional Review Board approval” [18]. Additional clinical information was obtained from the original authors’ submission, the soft file in entry GSE4573, and from supplementary data in the published paper. Matching of clinical cases to microarray samples was aided by using Unix scripts. The GDS expression data had been log transformed and normalized across the data sets for each Affymetrix probe. Density plots of each array revealed that the distribution of intensities was similar across the set and thus could readily be compared. The probe data set for each gene was averaged when multiple probes were present. In order to identify genes that were predictors of survival, gene subsets in which the interquartile difference was 0.5 logs or 1.0 logs, and in which > 0.25 of the log values were > 6.6 were chosen.

For the breast study, a total of 2874 HGU133A Affymetrix CEL files was obtained from GEO data sets GSE31519, GSE11121, GSE2034, GSE2990, GSE3494, GSE5327, GSE6532, and GSE7390, and the 98 of those that were triple negative cases were selected. These CEL files were processed using the rma function of the BioConductor affy library, and probes for the same gene were averaged. Since the files originated from multiple data sets, the data for each array were normalized to standard scores centered on zero using the scale function. These standard scores were used in the analysis. (However, similar results were obtained with the original scores.) The clinical data for 578 breast cancer cases were provided by the GSE31519 data set. These data were used to select a set of 63 cases that were suitable for logistic regression analysis of the early recurrence and long term, event-free survival groups. To select cases clinically similar to those used in the SQCC analysis, only patients with breast cancers classified as triple-negative, which carries a particularly poor prognosis [40], and who had not received adjuvant chemotherapy, were included.

The SQCC cases were first sorted based on given survival times, then the group of 20 earliest death cases was compared with the group of 20 longest survivors. In later analyses, groups of 20 from among the 40 longest survivors were compared to early death cases 1 through 20; conversely, groups of 20 from the 40 earliest deaths were compared to the longest 20 survivors. For each of the 80 comparisons, a logistic model for each of the 8,594 most variable genes was produced, and the accuracy of each model in predicting survival class was evaluated. Accuracy is the area under a ROC curve of 1 – specificity on the x axis and sensitivity on the y axis, where sensitivity is the proportion of true positive cases that are predicted correctly (sensitivity = TP/TP + FN where TP is the number of early death cases predicted correctly and FN is the number of long term survival cases predicted incorrectly), and specificity is the proportion of long survival cases predicted correctly (specificity = TN/TN + FP where TN is the number of long term survival cases predicted correctly and FP is the number of early death cases predicted incorrectly). It should be noted that the area under a ROC curve can be calculated by a simple, intuitive method, as described by Hosmer and Lemeshow [41]. Using this method, the ratios of each value in one class (early death group) with every value in the other class (longest survivor group) are calculated to determine how often the value in one class is less than or greater than the value in the other class. If, for example, 320 of the 400 ratios are greater than 1, the accuracy of that gene in predicting the correct class based on its expression values is 320/400 = 0.8. This ratio is precisely the area under the ROC.

The significance of each gene model was further evaluated using a chi squared ANOVA test of the logistic model slope coefficient, as described [41]. In the leave-one-out validation test, early death cases 5 through 24 were used to refine the gene selection; in our clinical experience, it is unlikely that at least the first four early postoperative deaths were related to SQCC progression.

## Results and discussion

**Figure 1:**
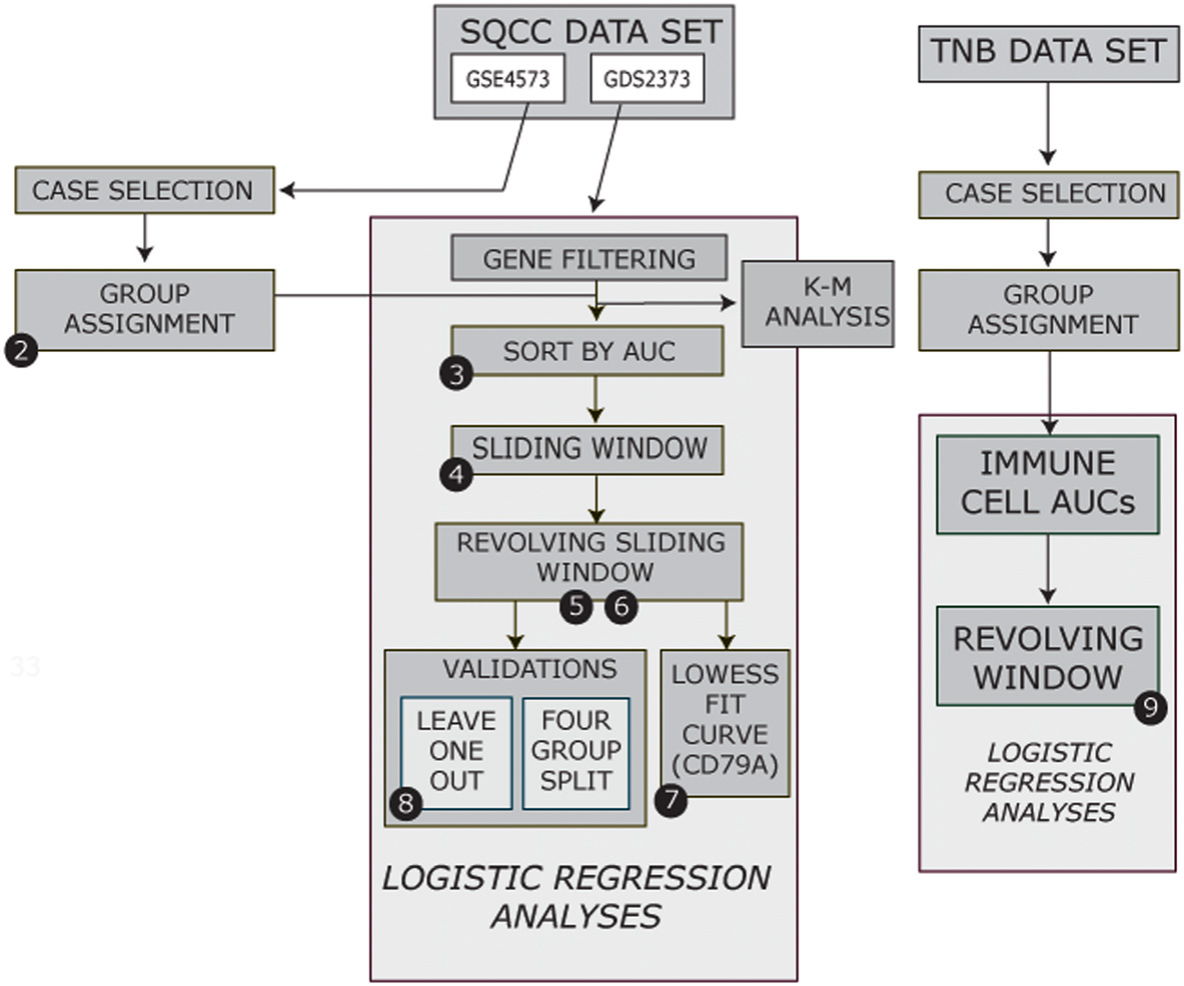
**Work flow of the logistic regression and ancillary analyses described in the Results and Discussion.** The numerals in black circles refer to the specific Figures related to that portion of the work flow.

The work flow of analyses described in this section is outlined in Figure 1.

### Data acquisition and case selection

Initially, we set as the aim of our statistical analyses the identification of individual gene expression changes prognostic of early death versus long survival in patients with stage I or II squamous cell carcinoma (SQCC) of the lung, a subset of patients in whom treatment choices are especially difficult [42]. We used a previously published data set (GDS2373, see Methods) of 130 primary SQCC samples from 129 patients, including 107 stage I and II cases and 23 stage III cases. Gene expression values were derived from tissue samples collected at the time of surgical resection and were analyzed using the Agilent U133A microarray platform [18]. The accompanying clinical data were obtained as described in Methods. The three published reports [18,20,21] of this data set included the 23 stage III cases. However, our analysis was limited to data from the 107 stage I and II cases, a selection consonant with the principle of using the clinical data in optimal fashion to achieve the objective of the study; limiting the cases to stages I and II provided a relatively homogenous patient sample in which the most prominent variable was survival.

**Figure 2:**
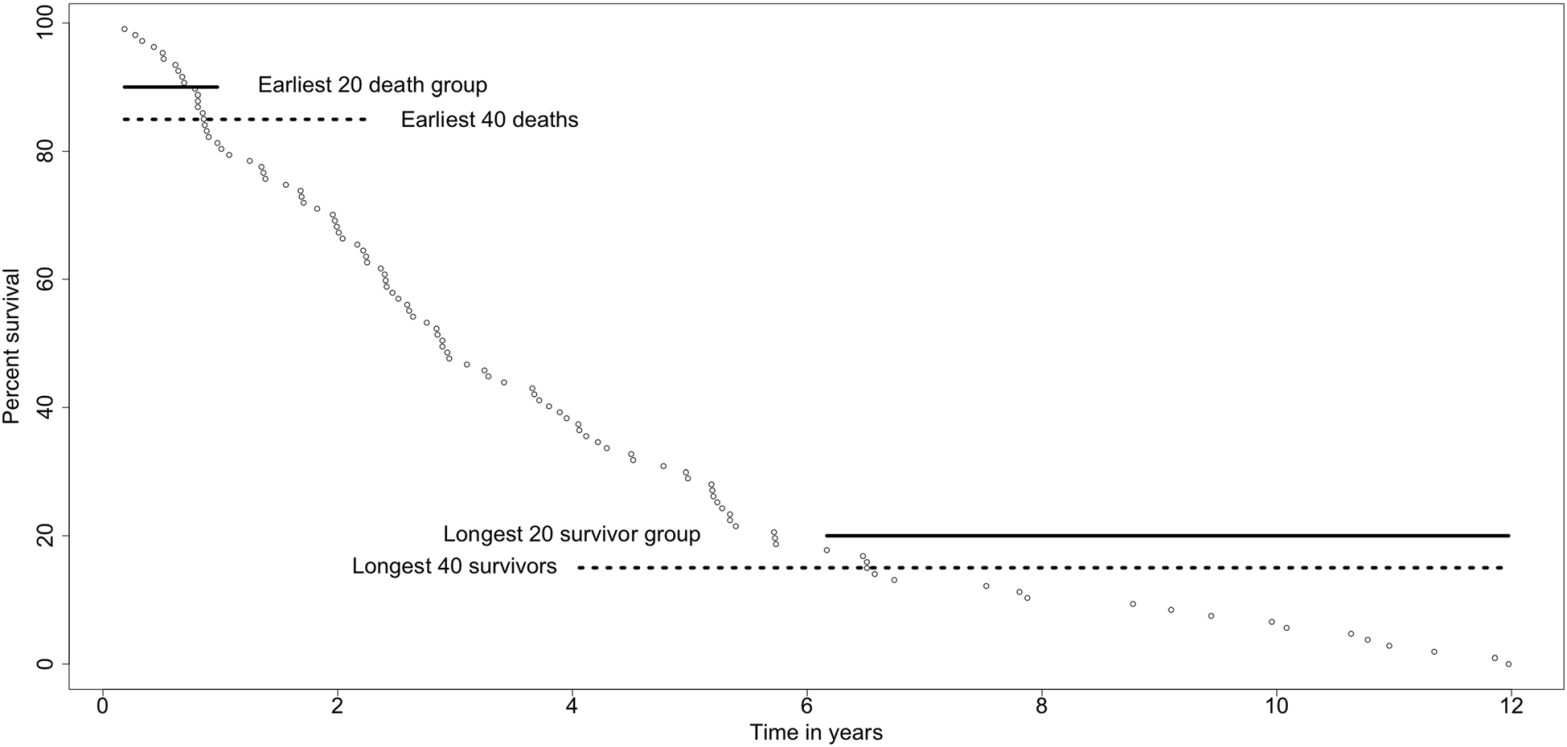
**The survival curve of stage I and II cases from the GDS2373 data set.** The curve is based on clinical stage data and durations of survival (see text) provided by the authors of the GEO data set. Also shown are the four groups used in our analyses, the earliest 20 or 40 deaths and the longest 20 or 40 survivors.

Application of the logistic regression method required two classes; we defined the two classes as those patients who died relatively soon after surgical resection (“earliest deaths”) and the ones who survived for a much longer time (“longest survivors”). The clinical data provided in the GEO author entry GSE4573 includes “duration of survival” calculated from the date of operation to the date of death or to the date of the last follow-up visit, if the patient was not known to have died. In the latter circumstance, the “duration of survival” represents the minimal survival time; actual survival for each of these cases is perforce longer, perhaps much longer. The plot in Figure 2 displays the fraction surviving as a function of the stated duration of survival [18]. Plotting survival by combined stage reinforces the homogeneity of the stage I and II cases. Also indicated in Figure 2 is the initial selection of two 20-patient groups of early deaths or long term survivors. Three criteria figured into the designation of the two groups: (1) a group size of 20 was chosen as suitable for comparisons involving only a single variable [41], namely survival; (2) equal sized groups are important to avoid model bias which occurs when one group is larger than the other [41]; and (3) all cases in the early death group were known to have died before two years and all of the longest survivors were alive at six years, even though four subsequently died. Thus, the compositions of the two groups of 20 cases were not affected by right-censoring. Also shown in Figure 2 are two larger groups of 40 cases each that were the basis for the sliding window analyses, used below; although a number of cases in the groups of 40 were right-censored, results consistent with these cases falling within the assigned survival groups were found.

Once assigned to a group, each case was considered comparable to all other cases in their group, without regard to the precise duration of survival. Doing so, which is possible because of the clinical homogeneity of the patient population under analysis, overcomes a major limitation of Kaplan Meier analysis, its dependency upon accurate survival data [43]. In many studies, the survival data are right-censored to varying degrees because of infrequent assessments and limited follow-ups. The logistic regression approach is less affected by incomplete or heavily right-censored survival data than KM analysis. An additional difficulty with analyses dependent upon durations of survival is that in the elderly population typical of SQCC, patient deaths not infrequently result from co-morbidities [44], such as infection, heart disease, stroke, emphysema and diabetes, rather than from cancer. Duration of survival, as in the KM method, is therefore an inadequate proxy for disease progression. Comparing groups of early deaths and long survivors minimizes errors introduced by limitations in the available survival data and by deaths not directly attributable to cancer progression. Similarly, in our method, the two groups were not defined by arbitrary time intervals, e.g., deaths within two years or survival greater than five years [45]; instead an equal number of cases was selected from either extreme of the survival spectrum.

### Initial prognostic gene selection by logistic regression

**Figure 3:**
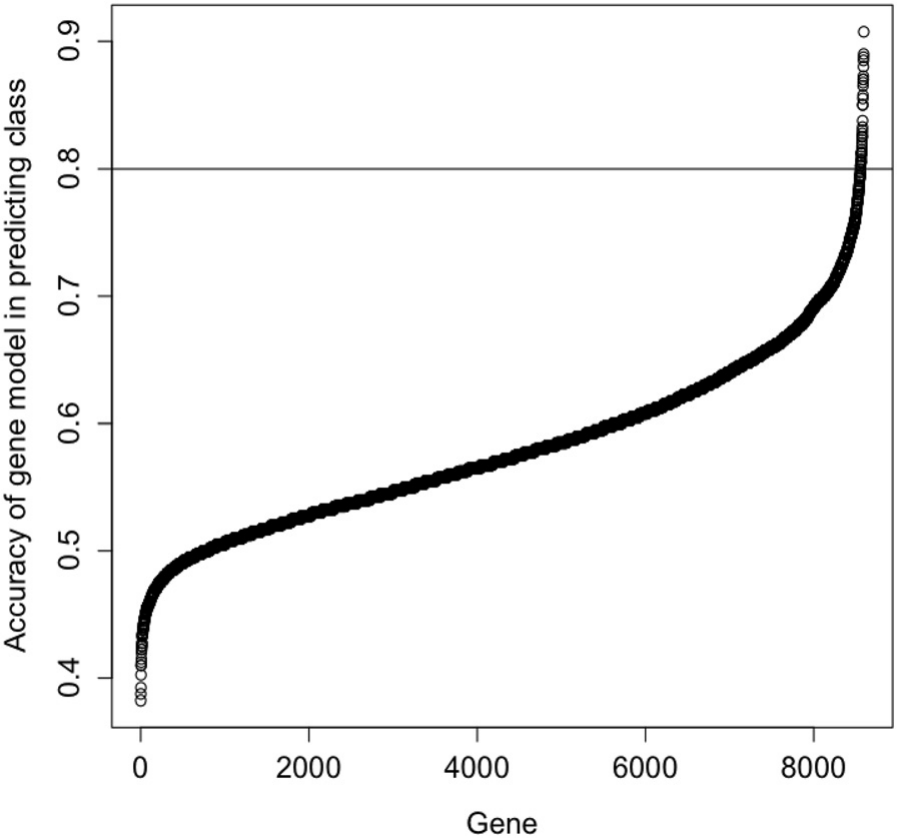
**A sorted plot of accuracies in predicting early death or long survival.** Using a logistic regression model of the stage I and II cases, each of the 8594 genes which varied at least four-fold across the entire GDS2373 data set was evaluated for accuracy in survival prediction. The accuracy is derived from the area under the ROC curve for each gene when comparing the earliest death group of 20 cases and the longest survivor group of 20 cases, which are shown in Figure 2.

Because the 12,704 gene expression values in data set GDS2373 are listed by gene and array ID, mapping of case ID to array ID (provided in file GSE4573) allowed the expression values for each case to be retrieved. First, in order to identify the most variable genes and reduce the influence of less variable ones, we applied a filter to the expression data to cull the gene list to the 8,594 genes which varied at least four-fold across the entire data set. Then, each of the 8,594 genes was subjected to logistic regression analysis to identify those genes which most accurately differentiated the early death group from the long survivor group. As a measure of accuracy of a particular gene, the area under the receiver operating characteristic (ROC) curve was calculated, see Methods. The curve in Figure 3 depicts all 8,594 genes sorted by their accuracies in predicting survival class. An accuracy value of 0.8 or greater is considered an excellent discriminatory model [41]; 40 genes fell within that range. The majority of the 40 genes appeared to be immune cell-related.

### Refinement of the prognostic gene list by sliding window analysis

**Figure 4:**
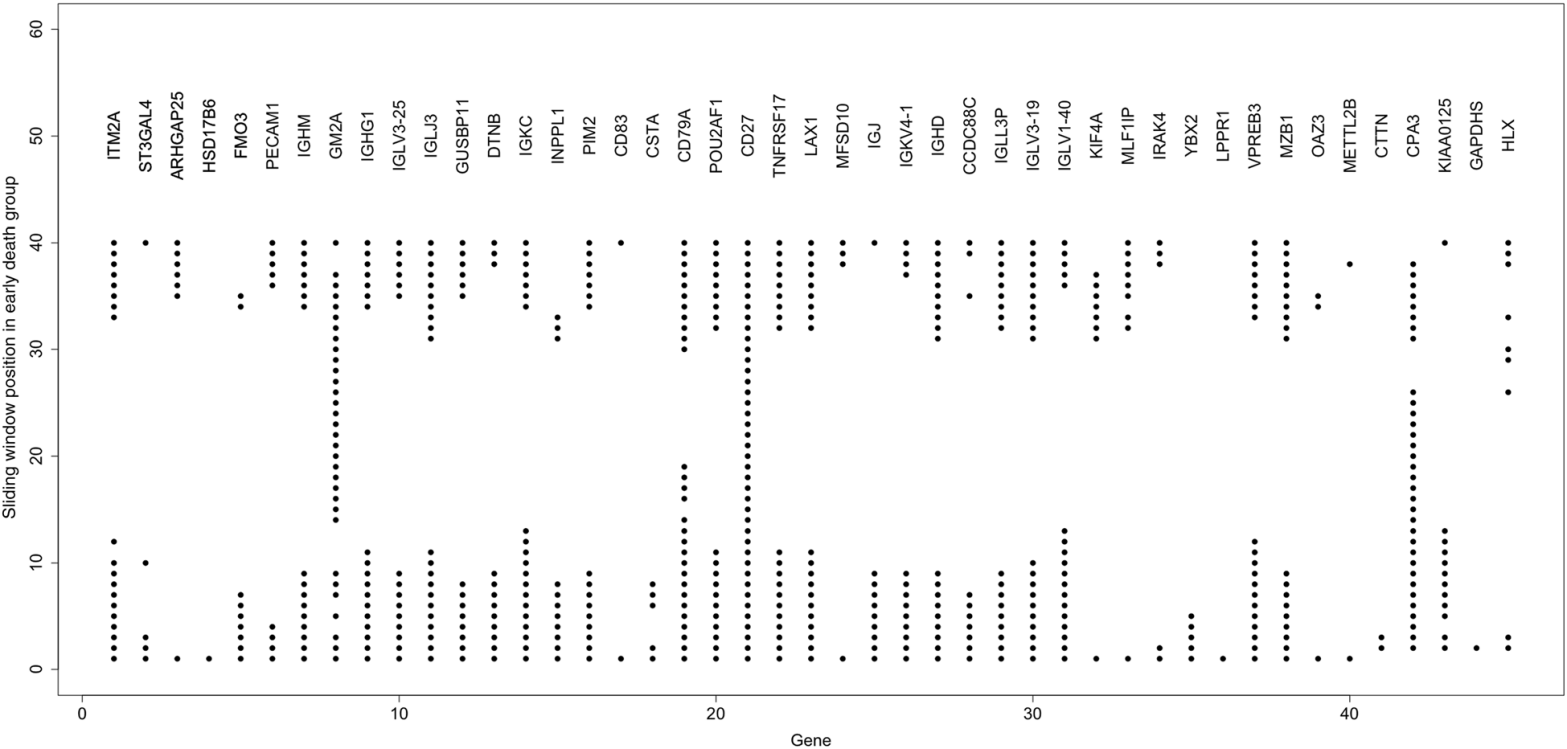
**Genes that predict early death or long survival with an accuracy of 0.8 or better.** Sequential sets of 20 early death cases (1 – 20, 2 – 21,…21 – 40) were compared to a constant set of the 20 longest survivors (cases 88 – 107). The Y-axis denotes each sequential window; each row of dots indicates the genes which were found to be predictive for that window. Shown on the X-axis are the 45 genes most frequently identified of the 99 genes found to be predictive in one or more windows.

Our list of genes predictive of survival was improved by two additional analyses. The first and simpler approach was to increase the number of comparisons per gene by creating 20 windows of early death cases by advancing the early death window one case at a time while holding the group of 20 longest survivors constant. The 45 genes most often found to achieve an accuracy of >0.8 as the early death window was advanced are shown in Figure 4. It was quite apparent that the same genes are often found regardless of the choice of the early death group, and that immune system genes remained strongly represented. The entire list of 99 genes is given in Additional file 1: Table S1. It should be noted that as the early death window advanced, greater numbers of right-censored cases were included. However, these cases also supported the same gene list.

**Figure 5:**
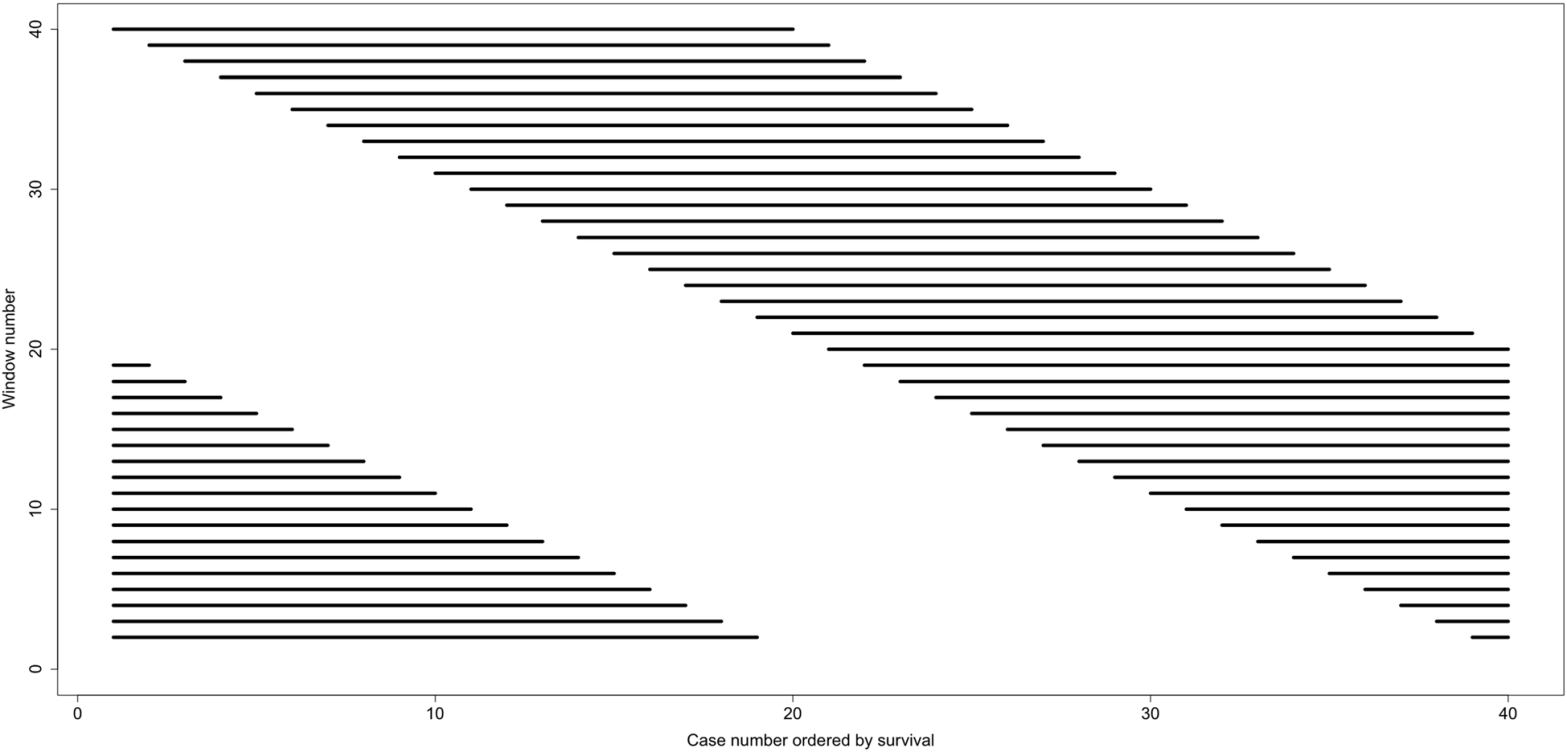
**The revolving sliding window method for selection of 40 consecutive sets of 20 cases each.** Shown here is the revolving sliding window for early deaths (the same approach was used with the 40 longest survivals). The first window selected is from case 1 to case 20, then from case 2 to 21, etc., until there are no longer 20 sequential cases available without going beyond the 40th case shown in Figure 2. At that point, the first earliest death case is used to complete the window of 20 and so on, using sequential cases. The 40 revolving sliding windows of early deaths or long survivors was compared to the opposing fixed group of 20 cases, also indicated in Figure 2. In total, 80 comparisons were made.

**Figure 6:**
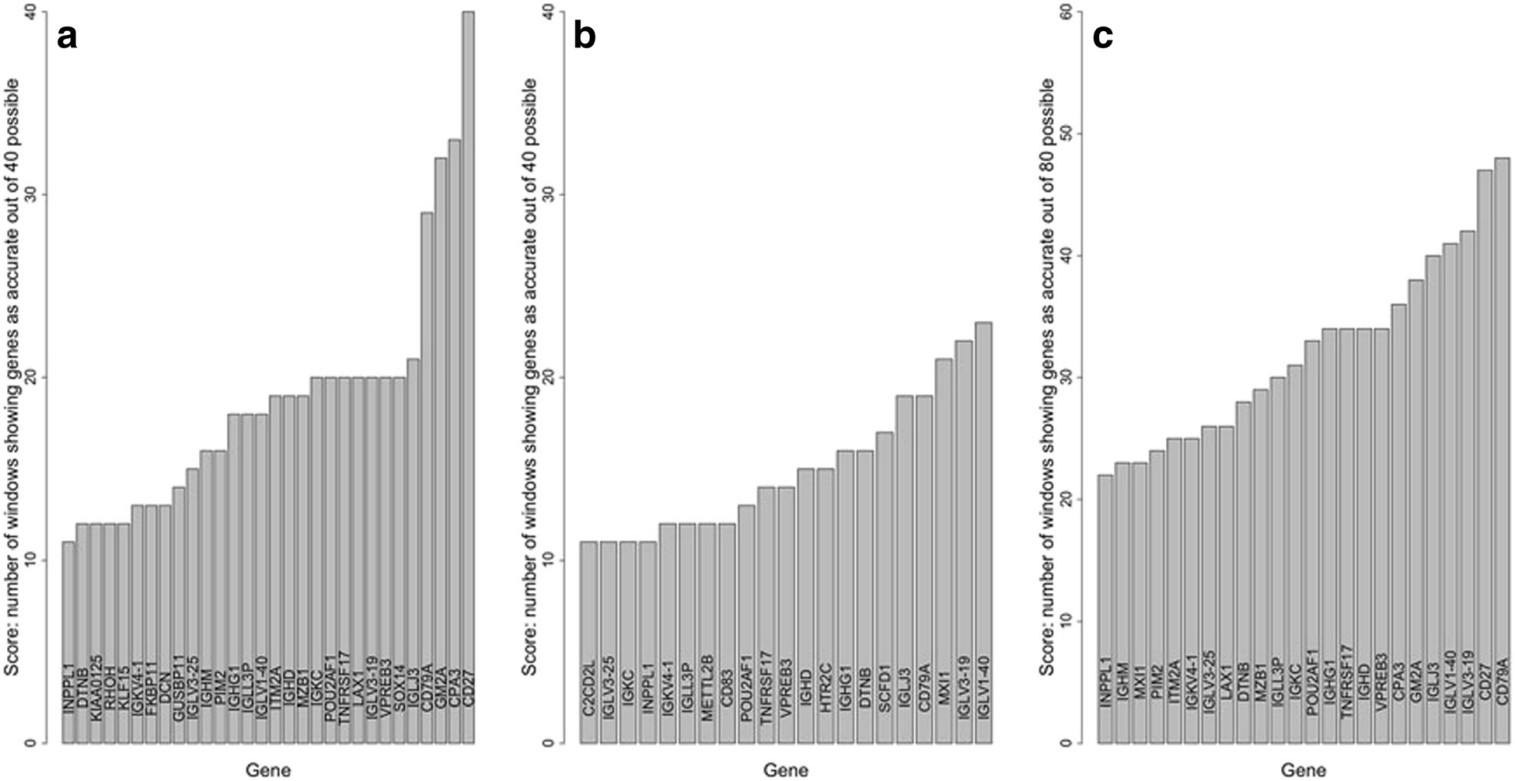
**Genes predicting early death versus long survival with the revolving sliding window method.** The scores shown on the Y-axis represent the number of windows in which an accuracy (area under a ROC plot) greater than 0.8 was achieved by logistic regression. The genes most frequently exceeding the 0.8 threshold are shown in order of numbers of occurrences out of the maximum possible score of 80, which is the total number of comparisons. **(a)** As shown in Figure 5, 40 sequential windows of 20 cases each were compiled from the early death group of 40 (Figure 2) and compared to a constant set of the 20 longest term survivors; thus, the maximum possible score was 40. Only genes which achieved a score > 10 of 40 are shown. **(b)** Revolving sliding windows of 20 long term survival cases each among the group of 40 longest survivors were compared to a constant set of 20 early deaths (Figure 2). Again, the 40 comparisons allowed a maximum score of 40. **(c)** Summation of the results from panels A and B. Because a total of 80 comparisons were made, the maximum possible score was 80. The two most accurate genes (CD79A and CD27) achieved scores approaching 50.

A drawback of the sliding window analysis just described is that cases clustered around the midpoint of the sliding window range are overrepresented whereas those at either end of the 40 case group were sampled less frequently (once for cases 1 and 40, twice for cases 2 and 39, *et cetera*). To circumvent uneven sampling, yet maintain the order of survival as much as possible, a revolving sliding window approach was applied, see Figure 5; in the collection of 40 windows so compiled, each case is sampled exactly 20 times. Each of the 40 early death windows so obtained was compared with a single, constant window of the 20 longest survivors. A list of genes that most often achieved an accuracy of >0.8 as the window was advanced was obtained. A barplot of the genes sorted by score of at least 10 out of a possible 40 windows is shown in Figure 6a. Then the process was reversed, comparing 40 revolving sliding windows compiled from the 40 longest survivor cases with a constant window of the 20 earliest deaths. A barplot of the list of genes sorted by score is given in Figure 6b. In both analyses, the constant window did not include right-censored cases of any significance, whereas the revolving sliding windows contained varying admixtures of right-censored cases. Combining the two revolving window analyses created a total of 80 opportunities for a given gene to obtain a ROC accuracy score > 0.8. A ranking of genes sorted by the number of times this score was achieved is depicted in Figure 6c; shown are the 24 genes which scored accurate hits in at least twenty of the 80 windows. Brief functional descriptions of the 24 genes are provided in Additional file 2: Table S2. By the revolving sliding window analysis, the most accurate prognostic genes were CD27 and CD79A which have scores approaching 50 out of the possible score of 80 (Figure 6c); however, every gene on the list provides excellent accuracy. The entire consensus list of 59 genes from this analysis is given in Additional file 3: Table S3.

Just as was found in the initial analysis, the majority of the 24 genes are immune system-related, especially reflecting B cell activity (Additional file 2: Table S2). Because the original tissue samples analyzed for the GDS2373 data set were limited to ones having a tumor cell population greater than 70% (Supplementary Information, [29]), it is unlikely that stromal cells surrounding the tumor biased the expression data. A second possibility which must be entertained is that the SQCC neoplastic cells themselves might express genes ordinarily assumed to be of immune cell origin, for example IgG [46,47]. We favor a third hypothesis: namely, that lymphocytes, especially B cells, had infiltrated the tumors to varying degrees, a well-documented phenomenon in solid tumors, as reviewed by Fridman, *et al.*[48].

**Figure 7:**
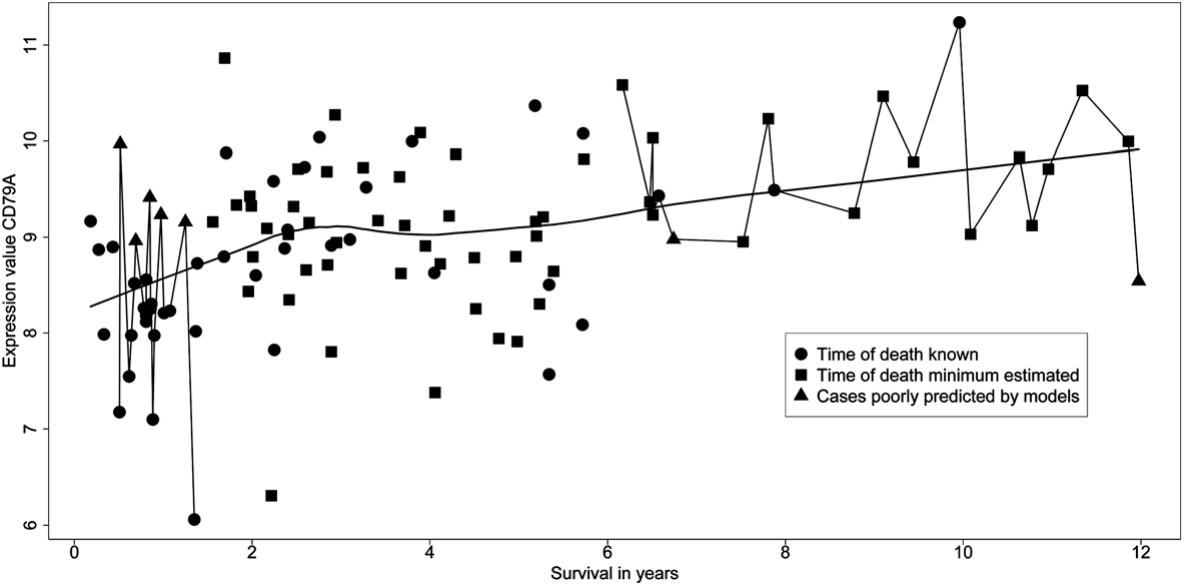
**Scatter plot of individual expression values of gene CD79A plotted against durations of survival.** Only stage I and II cases are shown; the survival data, including right-censored cases, are as given in GSE4573. Shown are data points that are based on time of death (filled circles) or right censored survival based on last clinical visit, shown as filled squares. The data points interconnected by lines are from the two groups of 20 cases (early deaths and long term survivors) which were used for the validation test shown in Figure 8. The smooth curve portrays a Lowess fit to all data points. Cases that were often not correctly predicted by the leave-one-out validation test in Figure 8 are also indicated (filled triangles). The durations of survival for these poorly predicted cases were all based on known time of death, except for the longest surviving case.

In order to gain additional insights into the use of logistic regression models for predictive gene identification, a plot of the expression values for the best prognostic gene, CD79A, as a function of duration of survival, is shown in Figure 7. The expression values for the two 20-patient groups (Figure 2) are joined as short scatter plots. The considerable variability of CD79A expression values suggests that the data are not suitable for analysis by any method which assumes a normally distributed variation. One reason for choosing the logistic regression approach is that normality of the data distribution is not required. Despite the variability in expression of CD79A, more than 80% of the values in the early death group are less than those in the longest survivor group. Also shown in Figure 7 is a moving linear regression line (LOWESS fit, locally weighted scatterplot smoothing [49]), which indicates that expression values increase with survival time. This observation adds further credence to the choice of CD79A as a prognostic gene. Finally, the expression values for certain cases, indicated by filled triangles, were in poor agreement with the CD79A gene model; these outliers consistently defied prediction [45] by most of the gene models, as illustrated below.

### Validation of the 24 prognostic genes

**Figure 8:**
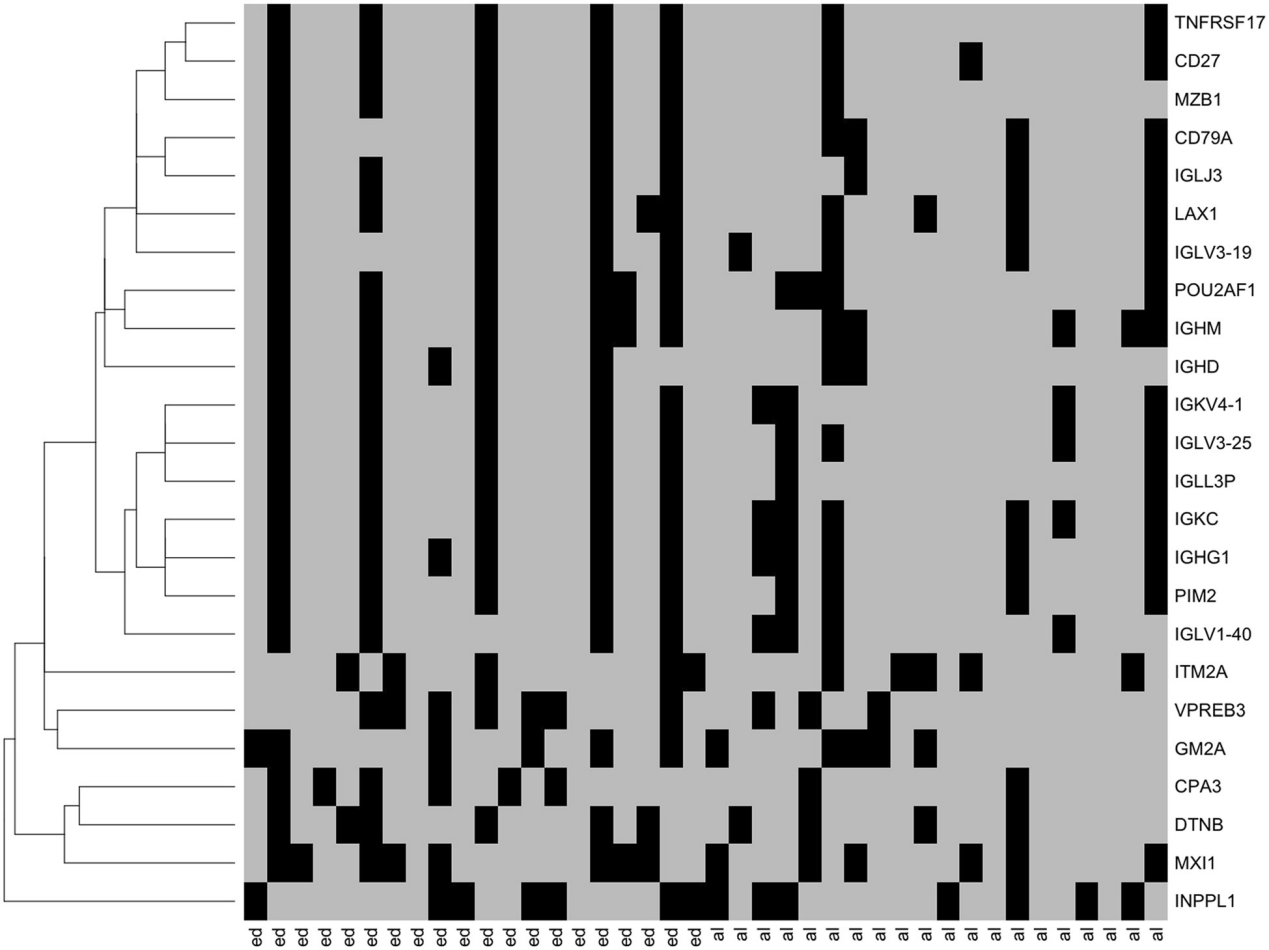
**Leave-one-out validation test of the 24 genes which best predict survival class.** Using the 20 early death and 20 long term survival cases indicated by the scatter plots in Figure 7, one case was left out and the remaining 39 cases were used to produce a logistic regression model which was then used to predict the left-out case; the process was continued sequentially for all 40 cases. Columns labeled “ed” are early death cases and columns labeled “al” are long term survival cases. Cases predicted correctly are shown in grey, incorrectly in black. The genes have been clustered based on similar class predictions.

In order to validate the prognostic genes identified by the revolving sliding window analysis, the data set was divided into model training and validation sets. A significant handicap when using small data sets for statistical modeling is the problem of over-fitting, which occurs when a model is validated using the same cases that were used to produce the model [10]. There are several ways to avoid this problem. One approach is to show that the originally derived model is predictive for survival in a second, entirely separate but comparable data set; the difficulties associated with finding and utilizing another suitable data set, discussed by others [50], were also experienced by us. A second approach is to divide the data into a model training set and a model validation set. We chose a variation of the latter, the “leave-one-out” method. For this analysis, constant windows of 20 early death cases (patients 5-24, cases 1 – 4 were censored, see Figure 8, legend) and 20 long survival cases (88-107) were selected, one of the 40 cases was left out, and the remaining 39 cases were used to predict the group in which the fortieth case resided; the process is repeated for each of the 40 cases.

The results of the leave-one-out analysis are shown in Figure 8. First, we ascertained that the most accurate genes correctly predicted 80 - 85% of early deaths and long survivals; however, even the two least accurate models (MXI1 and INPPL1) nonetheless predicted 65% of the cases correctly. Second, ANOVA chi-square tests were applied to each of the 24 logistic regression gene models and the range of probabilities for each was determined. These varied from 10^-4^ - 10^-3^ for MXI1 to 10^-8^ - 10^-6^ for CD79A (Additional file 2: Table S2), indicating, for example, an especially high level of confidence in the logistic model slope coefficient [41] for the latter gene. Models for the 17 genes in the upper portion of Figure 8 were likewise strongly supported by this analysis.

Finally, when the 24 prognostic genes were clustered based on their case-by-case predictions as shown on the left side of Figure 8, it was evident that five of the early death cases and two of the longest survivor cases were incorrectly predicted by most of the gene models. In a similar context, Zhao, *et al*. [45] have discussed the difficulty of predicting clinical outcomes from gene expression data in patients with rapidly progressive disease. Some of the uniformity of predictions – both accurate and erroneous - might be the consequence of disproportionate representation of certain cell types among the tissue samples [51] or might arise more directly from close functional relationships among the genes, hence an increased likelihood of coordinate gene expression. In support of the latter possibility, the cases which failed prediction by the CD79A model (indicated by closed circles in Figure 7) were consistent outliers; the same cases were incorrectly predicted by most gene models (Figure 8). Pearson correlation coefficients of the expression values for CD79A versus the other 23 genes were greater than 0.7 for 16 and greater than 0.8 for 10 genes, Additional file 4: Table S4. The gene expressions which did not correlate as well with CD79A are the lower six in Figure 8. Well-coordinated genes cannot be considered independent predictors of outcome. Nonetheless, the fact that so many immune-related genes were identified by each of our independent analyses supports their biological and functional relevance to survival. Hence, our data suggest that the strongest genetic signal for long-term patient survival in early-stage squamous cell carcinoma of the lung is an expression pattern reflective of increased number and/or activity of immune cells within the primary tumor.

As a more critical test of validation of the survival models, cases in the same early death and long survival groups (cases 5-24 and 88-107) were each divided into two groups of ten, using a set of every other case in each of the 4 groups. For example, test early cases 1,3,5,7,9,11,13,15,17,19 were used to predict the even numbered early cases, and this process was then reversed. A similar grouping and comparison was performed for the long survival cases - thus providing a total of four comparisons - and the accuracy of these predictions was determined. For two of the best predictive genes in the leave-one-out analysis, CD79A and CD27, their average accuracies in the four-group comparisons were 0.76 and 0.78, respectively, thus further validating the prognostic value of these 2 genes.

One alternative to these approaches is to randomly and repeatedly select groups of 20 patients from the 40-case earliest death and longest survival groups in a bootstrap or resampling type of analysis, and collect a list of most predictive genes. The bootstrap method may be more appropriate if patient survival is not as accurately specified as in the GDS2373 data set or if there are other clinical variables that may be a factor in choosing predictive genes. Additional resampling approaches have been discussed by others [52,53].

### Logistic regression versus Kaplan Meier analysis

The list of 24 prognostic genes identified by logistic regression was also compared to a list of genes obtained from the same data set using the more conventional approach of KM plots of expression quantiles. Initially, the 8594 most variable genes were tested as predictors of survival for the 107 stage I and II cases using right-censored survival for each case and the chi square statistic as a test of equality between four quantiles. Fourteen of the 24 genes found by the logistic regression method were also present in the list of the 40 best scoring genes (*P* < 10^−3^) by KM analysis (Additional file 5: Table S5) and five (IGLJ3, IGKC, IGHD, GM2A, DTNB) were in the top ten. The functions of the remaining genes found by KM analysis did not appear to be related to the immune system.

To more closely compare the two methods, a similar KM analysis was also performed using the same 40 cases that were used for the logistic regression analysis shown in Figure 8. Nine of the top 24 genes found by the logistic regression method (IGHM, GM2A, DTNB, INPPL1, CD27, TNFRSF1, LAX1, IGKV4-1, IGHD) were in the list of 24 best scoring genes (*P* < 10^−4^) by this modified KM analysis, whereas the remaining 15 were not apparently related to the immune system. Four of these genes were the highest scoring ones (*P* < 10^−5^, genes GM2A, INPPL1, CD27, and IGHD) by KM analysis. Thus, the KM method used with all 107 stage 1 and 2 cases, or with a reduced set of 40 early death and long term survival cases, also revealed that a set of immune genes are strongly predictive for survival. Finding similar sets of immune-related genes by the KM and logistic regression methods, which use different computational approaches provides additional confirmation that these genes are reliable predictors. This result also extends the validation analysis of the logistic regression models performed in Figure 8. The two methods contrast in that the KM method predicts a survival curve based on the quantile rank of a gene expression value, whereas the logistic regression method predicts a survival class (early death within two years or long survival greater than six years) for a given gene expression value.

That the GDS2373 clinical data included a preponderance of accurate survival times with long follow-ups undoubtedly contributed to the sensitivity of the KM method in this instance. Ordinarily, patient survival data is derived from a censoring analysis in which the survival time of each patient must be estimated and often, many of the cases have limited follow-ups spaced at longer intervals. As the intervals between censoring assessments increase and their numbers decline, the sensitivity of the KM method decreases [54]. In contrast, the logistic regression method described here only requires of the survival data that two approximately equal-sized groups can be chosen from opposing extremes of the survival spectrum; these groups can be identified with a relatively small number of assessments of patient survival.

One theoretical limitation of the logistic regression method, however, is that by choosing groups at the survival extremes, not all cases in the data set are included in the analysis. In fact, 80 (75%) of the 107 available stage I and II cases were used in our analysis. Moreover, the intermediate survival cases, which are heavily right censored and may thus degrade the analysis, are of lesser significance for predicting survival class and need not be used. The experimental objective articulated in the original analysis of this data set by Raponi, et al., [18] was to identify gene profiles that influenced the duration of survival, whereas our logistic regression method was designed to identify genes predictive of a survival class. The latter objective simplifies the experimental design and allows less frequent assessments of survival; thus for clinical studies it may be more practical and less expensive.

In all three of the previously reported studies [18,20,21] of the GDS2373 data set, stage III cases were included in the KM survival analyses. Of the 112 genes identified as prognostic in the three studies, only four appear on our 24 gene list. Consequently, we repeated our KM analysis with all 130 cases, including the 23 stage III cases. Only two (INPPL1 and GM2A, which are perhaps not immune-related, Additional file 2: Table S2) of the 24 genes found by the logistic regression method were present among the 40 top scoring genes (4*10^−5^ > *P* < 1.4*10^−3^) found by KM analysis. Many of the remaining 38 (data not shown) were tumor-related genes commonly identified in such studies (e.g., KRT7, VEGFA). An obvious but important conclusion is that immune system genes are identifiable by conventional KM analysis only when the expression data are limited to stage I and II cases. As a further comparison to KM methodology, the logistic regression analysis was repeated but this time including the stage III cases in the data set. Doing so changed the compositions of the 20-case early death and long survival groups with the consequence that immune system genes were less prevalent in the most predictive gene set (data not shown). These differences are not unexpected as the more advanced stage III tumors almost certainly have undergone additional genetic changes [2], which in turn influence their expression profiles, likely overwhelming the immune cell contributions to the gene expression pool. Also, rapid proliferation and attendant necrosis of cells within stage III primary tumors may alter lymphocyte to tumor cell ratios [55], again decreasing relative B cell gene expressions.

Although our KM analysis did identify some immune-related genes as prognostic, the logistic regression approach proved superior in that it identified a larger number of highly correlated B cell genes in the stage I and II cases of the GDS2373 data set. Importantly, with logistic regression, one can increase the number of comparisons for each gene model by using sliding and revolving windows of early death and long survival cases, providing additional evidence in support of the prognostic gene list. Our results with logistic regression (and, for that matter, with KM analysis) also demonstrate the essentiality of stratifying the available clinical data commensurate with the study objective in order for the prognostic gene profiles obtained to be of potential clinical value [10]. These results also underscore the importance of using clinical data appropriately to achieve a more informative statistical analysis [10]. As mentioned earlier, stage I and II cases present difficult therapeutic decisions [42]. Somewhat less than half of the patients will ultimately die of disease progression [56] and therefore should be treated aggressively; however, if every patient is so treated, the majority will suffer the adverse consequences of therapy unnecessarily. Thus, for stages I and II accurate prognostic information complementary to staging will improve therapeutic decision making [42,57].

### Application of the logistic regression method for predicting clinical outcome in a triple negative breast carcinoma (TNB) data set

An immune cell signature has also been found to be predictive for clinical outcome in triple negative breast carcinoma [58]. In the published study, clustering of genes with respect to time of first event (recurrence of the tumor) against gene expression values revealed a group of genes that included immune-related genes. The median gene value of this set was then used in Cox proportional hazard models with clinical variables and KM plots to reveal an influence of immune cell expression on outcome. Because of the clinical similarities of TNB and SQCC with respect to rate and timing of recurrence in early stage cases, we also applied our logistic regression approach to a TNB subset of their data set.

For our logistic regression analysis we selected a group of 63 triple negative breast cancer cases (see Additional file 6: Table S6 for the list of CEL files) from the supplementary data of the original report. The cases selected had complete clinical data, and were early stage lesions classified as T1, N0 malignancies (and tumor grades 1, 2 or 3). All patients included in the long term survival group were event-free at the time of the last follow-up visit. Of the 63 TNB cases, 31 had first events (recurrence of the tumor) within 18 months and 32 were event-free ten years after tumor removal. From 63 triple negative breast cancer cases, a group of 20 cases with the earliest recurrence of the tumor and a second group of 20 cases that had not experienced tumor recurrence for the longest duration were selected. Each gene in the normalized data set was then subjected to a logistic regression analysis and the area under the ROC curve (AUC) determined. Less variable genes were not filtered out as was done for the SQCC data in order to capture the full extent of involvement of the selected genes. AUC values for a set of immune related genes within the data set were then determined. A total of 203 immune-related genes represented on the HGU133A microarray were found using the search terms “immuno”, “lymph”, “B-cell”, and “T-cell”, and by adding 20 of the 24 genes found in the lung study. The list of genes and the AUC values are given in Additional file 7: Table S7. Three of the genes had AUC values > 0.8, 19 genes greater than 0.75, and 45 genes greater than 0.7. The two top-scoring immune genes were BANK1 (AUC = 0.86) and BLNK (AUC = 0.8), which encode a B-cell scaffold protein and a B-cell linker, respectively. A significant difference of the distribution of AUC values between all genes and the sample of 203 immune related genes was also found (*P* < 0.0016, by Kolmogorov-Smirnov test). There were just three non-immune related genes with AUC values greater than the most predictive immune gene (AUC > 0.86); this list is provided in Additional file 8: Table S8.

**Figure 9:**
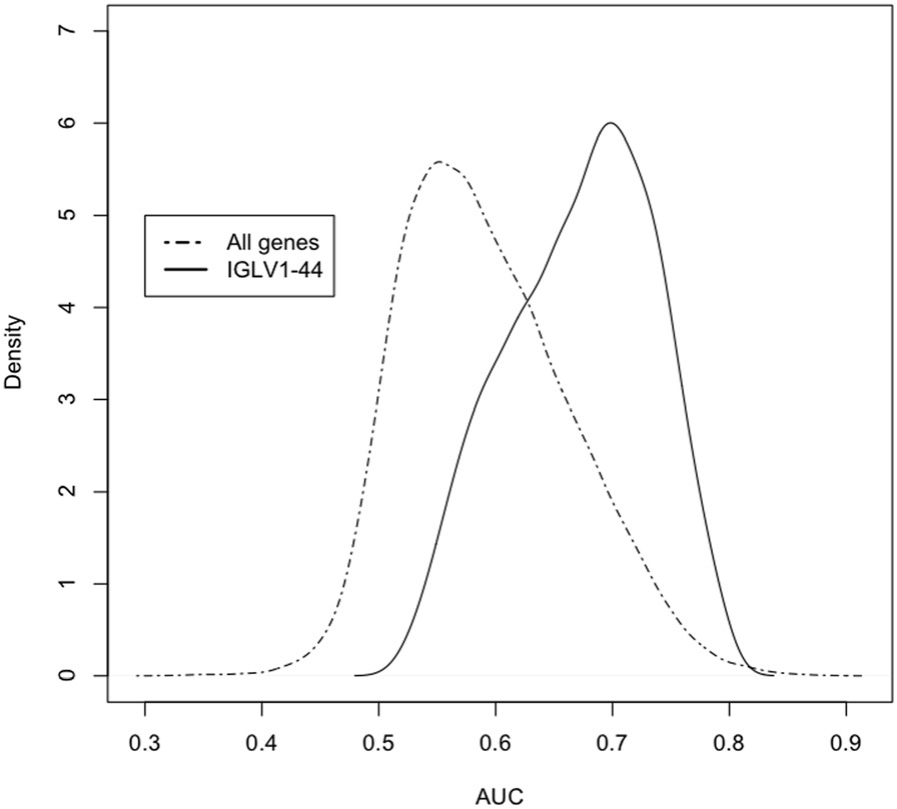
**Distribution of AUC values for gene ILV1**-**44 produced by the revolving sliding window method compared to all genes in the TNB data set.** A set of 30 windows of length 20 were produced by the revolving window method for the first 30 and last 30 cases in the breast cancer data set, representing earliest events versus longest recurrence-free survival, respectively. Each of the 900 possible combinations were analysed to produce an accuracy score (AUC). Shown is the distribution of scores for one of the most predictive genes, ILV1-44, in comparison to the distribution curve for all other genes. The plot is representative of the AUC analysis for the other immune genes found to be predictive in the TNB study.

To obtain further evidence these results are independent of the cases chosen, a modified revolving window approach was performed on the breast data similar to that used in the analysis shown in Figure 6. A set of 30 consecutive windows of length 20 was generated in each group of patients. Nine hundred comparisons were then made and the distribution of AUC values obtained. A density distribution of these values for one of the best predictive genes, ILV1-44, original AUC = 0.78, is shown in Figure 9. The plot reveals the variation in the data set and also illustrates that the distribution of scores is significantly greater for the ILV1-44 (immune) gene model than in the entire gene set (*P* < 2.2*10^−16^ by Kolmogorov-Smirnov test). A similar test was applied to the genes with the highest AUC values (AUC > 0.7) and the *P* values were highly significant for all except gene BLNK. The values for many of the additional immune genes set are highly correlated (Pearson correlation coefficient > 0.65) with those of IGLV1-44 and their AUC distributions are also expected to be significantly above normal. These correlation values are given in the supplementary data (Additional file 9: Table S9). These results indicate that the logistic regression method can also detect an immune signature in the triple negative breast carcinoma data set. Immune-related genes, however, were not all found at the top of the list as was found in the lung data set, although they do rank highly in the overall distribution of AUC scores; only a relatively small number of genes are better predictors of recurrence outcome. The lung and breast diseases are different and one can reasonably expect that different genes will influence clinical outcome. Moreover, the clinical endpoints in these two studies, survival and disease recurrence, are different. Remarkably, clinical outcome in the early stages of both diseases is particularly influenced by B cell gene expression, suggesting a biologic role for immune B cells in both of these cancers.

### The role of B cells in early-stage SQCC of the lung and triple negative breast cancer

Numerous reports have analyzed immune cell, especially T cell, responses to malignancies (reviewed by Whiteside [39] and Prado-Garcia, *et al*. [59]). Recently however, attention has been drawn to B-cell gene expressions, as indicative or suggestive of improved survival, in various solid tumors [60], including NSCLC, as reviewed by Suzuki et al., [61]; adenocarcinoma [61], small cell [62], and large cell [63] carcinomas of the lung; breast cancer [64,65]; and colorectal carcinoma [60]. Prognostic B cell gene expressions in patients with solid tumors have also been documented in analyses of regional lymph nodes [66] and peripheral blood mononuclear cells [67,68]. The role of immune cell-related genes, especially those of B cell origin, as prognostic of SQCC survival, has likewise been suggested previously. Roepman, et al., in a 72-gene classifier derived by Cox proportional hazards models from a 172 NSCLC patient data set (of which 53% were SQCC cases), identified a number of immune-related genes, about 20% of their 72 gene list [19]. As in our analysis, the patients in their study were limited to stages I and II and did not receive adjuvant therapy.

Similarly, we have identified numerous immune-related genes as prognostic in triple-negative breast cancer. Although not a novel finding per se, the clarity of the observations suggests that as with SQCC of the lung, TNB cancers should be scrutinized further to better define the role of immune cells in preventing recurrence.

Genome sequencing of tumors has led to the realization that mutations in a relatively small number of driver genes promote tumor development by influencing only a few key signaling pathways, which in turn affect cell survival, cell fate or genome maintenance [2]. Nearly all solid tumors in adults carry, in addition to driver mutations, appreciable numbers of mutations which do not confer a growth advantage; non-small-cell-lung-cancers are especially rich in these passenger mutations because of exposure to carcinogens [2] before and during tumor cell development. Many of the mutations, of driver and passenger genes alike, can be presumed to influence the gene expression profile of each lung cancer cell, adding to the difficulty of finding common gene profiles; the signal of cancer-related changes must be found against a large, variable background of noise. This background may explain the difficulty in obtained reproducible profiles of genes affecting survival when tissues from different studies are used.

The present study does *not*, in fact, report conserved tumor cell profiles but rather expression patterns that suggests the presence - among malignant cells of the primary tumor - of immune cells constituting a highly conserved defense system against neoplastic cells. The importance of this defense system is underscored by our observation that immune cell, especially B cell, expressions are greater in nearly all of the SQCC long survivors, compared to the early deaths, of the stage I and II cases in this study. Kawano, et al. [69] and Rena, et al. [70] have reported that up to 25% of stage I NSCLC patients in their studies were found to have isolated tumor cells or micrometastases when regional lymph nodes (RLN) removed contemporaneously with tumor resection were carefully examined by immunohistochemistry. However, survival rates were no different in the patients with RLN micrometastases, suggesting that host immune defense responses play a determinant role in the early phase of the disease [66]. The presence of this defense system has been reported previously but has probably more often escaped detection in gene expression analyses, in large part because of inappropriate use of clinical data and the application of less satisfactory analytical methods [10].

Based upon our application of the logistic regression strategy to the GDS2373 data set, as well as the corroborating observations cited above, we suggest that B cell function within the primary tumor may be an important prognostic indicator for stage I and II cases of SQCC. This conclusion warrants further study, for example, by analyzing comparable tumor samples for B cell gene activity using immunohistochemical methods or RT-PCR, in conjunction with accurate, non-censored survival data. Given the apparent activity of B cells in early-stage SQCC, NSCLC, and other solid tumors, one critical role for these cells might be recognition of tumor-specific antigens. Then, recruitment of T cells to tumor sites and/or occult metastatic foci and the destruction of tumor cells by humoral antibodies and lymphocytes could interface to dictate survival. It has been suggested that over-expressed genes, and specifically their protein and carbohydrate products, by neoplastic cells could be the source of such recognition [71]. Further analysis of expression data, supported by immunochemistry, may result in identification of additional candidate tumor-specific antigens [65,72-74].

## Conclusions

The many large gene expression data sets available in the public domain afford invaluable opportunities for analysing and understanding the effects of genetic and epigenetic effects on cellular phenotypes dictating outcomes in patients with malignancies. In this report we describe a logistic regression methodology for data set analysis which circumvents the principal shortcoming of conventional Kaplan Meier approaches, its reliance upon accurate survival data. Comparing classes of cases allows inaccurate, incomplete survival data to be used effectively. No less important is the careful stratification of cases based on clinical data and the choice of classes for comparison.

Our logistic regression analysis of a previously thrice-analysed SQCC data set revealed a number of B cell immune-related genes, all highly correlated in expression. This represents a novel finding in SQCC, although similar gene lists have been reported for other solid tumors. Indeed, we have also identified the predictive value of B-cell gene expressions in TNB. We propose that B cell activity within primary SQCC tumors is an important indicator of prolonged survival and, as such, merits further examination and experimentation. Understanding the role of B cells in determining outcomes in patients with SQCC may lead to improvements in diagnosis and therapy of this aggressive carcinoma.

## Abbreviations

ROC: Receiver operating characteristic; SQCC: Squamous cell carcinoma (of the lung); KM: Kaplan Meier analysis; AUC: Area under the curve (of a ROC plot); ANOVA: Analysis of variance; NSCLC: Non-small cell lung cancer; RLN: Regional lymph nodes; TNB: Triple negative breast carcinoma.

## Competing interests

The authors declare that they have no competing interests.

## Authors’ contributions

DWM conceptualized and designed the project and its methodology, analyzed and interpreted data, and participated in the drafting of the manuscript. CWP contributed to the design of the project, use of clinical data and group selection, interpretation of results, and drafting and editing of the manuscript. SMC assisted in the interpretation of the gene lists. AMM contributed to the statistical analysis of the logistic regression models. RP acquired data sets and assisted in their analysis. LLG contributed to the conceptualization of the project and the interpretation of clinical data. JDM participated in the conceptualization and design of the project, contributed to the interpretation of data, and participated in the drafting and editing of the manuscript. All authors have read and approved the final manuscript.

## Pre-publication history

The pre-publication history for this paper can be accessed here:

http://www.biomedcentral.com/1755-8794/7/33/prepub

## Supplementary Material

Additional file 1: Table S1 Genes identified in the first sliding window analysis. Click here for file

Additional file 2: Table S2 The 24 most accurate prognostic genes emerging from the logistic regression analysis of 80 revolving sliding windows. Click here for file

Additional file 3: Table S3 The entire list of consensus prognostic genes identified by the revolving sliding window approach. Click here for file

Additional file 4: Table S4 Correlation coefficients of CD79A versus the remaining 23 prognostic genes. Click here for file

Additional file 5: Table S5 Forty genes identified by KM analysis of stage I and II cases from the GDS2373 data set. Click here for file

Additional file 6: Table S6 A list of 63 Affymetrix HGU133A CEL files used for the TNB study. Click here for file

Additional file 7: Table S7 TNB logistic regression analysis: AUC of 203 immune related genes. Click here for file

Additional file 8: Table S8 TNB logistic regression analysis: 3 apparently non-immune related genes that have an AUC > 0.86, the best AUC score obtained with an immune gene. Click here for file

Additional file 9: Table S9 TNB logistic regression analysis: immune genes that are closely correlated with gene IGLV1-44 in the initial TNB data analysis; also given are the AUC values for each of the genes. Click here for file
