# Supplementary material for: Using logistic regression to improve the prognostic value of microarray gene expression data sets: application to early-stage squamous cell carcinoma of the lung and triple negative breast carcinoma
Source: BMC Med Genomics. 2014 Jun 10;7:33. doi: 10.1186/1755-8794-7-33 (PMC4110620; doi:10.1186/1755-8794-7-33)
Supplement: Additional file 1: Table S1 — Genes identified in the first sliding window analysis. [file 1755-8794-7-33-S1.pdf]

Table S1. Genes identified in the first sliding window analysis. A total of 99 genes were found. The accuracy count represents the number of windows in which each gene appeared.

NUMBER	GENE NAME	ACCURACY COUNT
1	ACSBG2	2
2	ARFIP1	3
3	ARHGAP25	1
4	ARID5B	2
5	C1S	5
6	C2CD2L	5
7	CC2D1A	1
8	CCDC88C	7
9	CCL2	3
10	CD27	20
11	CD79A	18
12	CD83	1
13	CHKA	4
14	COL3A1	2
15	CPA3	19
16	CSTA	5
17	CTTN	2
18	CYBA	2
19	DCN	12
20	DDR2	2
21	DEF6	8
22	DTNB	9
23	EGR2	2
24	ENO2	4
25	FAM46C	5
26	FBLN1	1
27	FBLN5	2
28	FCRL2	4
29	FKBP11	5
30	FMO3	7
31	GABRB2	1
32	GAPDHS	1
33	GM2A	14
34	GUSBP11	8
35	H2AFY2	1
36	HLX	2
37	HOXC8	1
38	HSD17B6	1
39	IGHD	9
40	IGHG1	11
41	IGHM	9
42	IGJ	9
43	IGKC	13
44	IGKV4-1	9

Table S1. Genes identified in the first sliding window analysis. A total of 99 genes were found. The accuracy count represents the number of windows in which each gene appeared.

45	IGL@	4
46	IGLJ3	11
47	IGLL3P	9
48	IGLV1-40	13
49	IGLV3-19	10
50	IGLV3-25	9
51	INPPL1	8
52	IRAK4	2
53	ISG20	3
54	ITGB7	6
55	ITM2A	11
56	KCTD3	1
57	KIAA0125	11
58	KIF4A	1
59	LATS1	2
60	LAX1	11
61	LPPR1	1
62	LRRC23	4
63	METTL2B	1
64	MFSD10	1
65	MLF1IP	1
66	MMP1	1
67	MMP17	3
68	MS4A1	1
69	MXI1	2
70	MZB1	9
71	NELL2	2
72	NEUROG1	3
73	OAZ3	1
74	PARM1	1
75	PDGFRL	5
76	PDZRN3	2
77	PECAM1	4
78	PIM2	9
79	PLA2G10	1
80	PLEKHB1	1
81	PNOC	5
82	POSTN	1
83	POU2AF1	11
84	PRDM1	6
85	PRNP	1
86	PTPLAD1	1
87	PTX3	1
88	RHOH	4
89	SLC19A1	2

Table S1. Genes identified in the first sliding window analysis. A total of 99 genes were found. The accuracy count represents the number of windows in which each gene appeared.

90	SLC35A3	1
91	SNUPN	2
92	SOX14	1
93	SPAG6	5
94	ST3GAL4	4
95	TNFRSF17	11
96	VEGFA	1
97	VPREB3	12
98	WNT2	3
99	YBX2	5
