# Supplementary material for: Using logistic regression to improve the prognostic value of microarray gene expression data sets: application to early-stage squamous cell carcinoma of the lung and triple negative breast carcinoma
Source: BMC Med Genomics. 2014 Jun 10;7:33. doi: 10.1186/1755-8794-7-33 (PMC4110620; doi:10.1186/1755-8794-7-33)
Supplement: Additional file 2: Table S2 — The 24 most accurate prognostic genes emerging from the logistic regression analysis of 80 revolving sliding windows. [file 1755-8794-7-33-S2.pdf]

Table S2. The 24 prognostic genes emerging from the logistic regression analysis of 80 revolving sliding windows.

GENE SYMBOL	GENE NAME	CHI-SQUARED PROBABILITIES[1]		CELL OR TISSUE ORIGIN	RELATIVE EXPRESSION IN LONGEST SURVIVORS[2]	IMMUNE OR NEOPLASTIC (ITALICS) FUNCTIONS
		LOW	HIGH			
CD79A	CD79a molecule, immunoglobulin-associated alpha	2.1e-08	6.1e-07	B cells, lymphoid cells.	↑	B-cell antigen receptor complex.
CD27	CD27 molecule	8.0e-07	2.6e-05	T cells.	↑	TNF superfamily receptor for CD70/CD27L.
IGLV3-19	Immunoglobulin lambda variable 3-19	1.5e-08	1.4e-06	B cells	↑	Ig lambda variable light chain.
IGLV1-40	Immunoglobulin lambda variable 1-40	1.0e-06	2.8e-05	B cells	↑	Ig lambda variable light chain.
IGLJ3	Immunoglobulin joining lambda 3	7.2e-08	2.4e-06	B cells	↑	Ig lambda joining light chain.
GM2A	GM2 ganglioside activator	3.1e-05	4.5e-04	Epithelial cells	↑	*Endosomal glycolipid transport and degradation protein.*
CPA3	Carboxypeptidase A3 (mast cell)	9.5e-08	2.3e-06	Mast cells, other tissues.	↑	Metalloexopeptidase complexes in secretory granules.
VPREB3	Pre-B lymphocyte 3	3.4e-06	7.9e-04	B-cell precursors.	↑	Biosynthesis of pre-B cell receptors.
IGHD	Immunoglobulin heavy constant delta	6.3e-07	9.4e-06	B cells.	↑	Heavy chain of IgD, B cell membranes.
TNFRSF17	Tumor necrosis factor receptor superfamily, member 17	2.3e-07	1.7e-06	Mature B cells.	↑	B cell receptor for TNFSF13B.
IGHG1	Immunoglobulin heavy constant gamma (G1m Marker)	6.3e-07	7.8e-06	B cells, carcinoma cells.	↑	Circulating immunoglobulins.
POU2AF1	POU class 2 associating factor 1	4.5e-06	4.6e-05	B cells; lung, various tissues,	↑	Transcriptional coactivator of OCT1 and OCT2.
IGKC	Immunoglobulin kappa constant	1.4e-06	1.3e-05	B cells.	↑	Constant domain of Ig kappa light chain.
IGLL3P	Immunoglobulin lambda-like polypeptide 3, pseudogene	3.9e-07	3.1e-06	N.A.	↑	Pseudogene.
MZB1	Marginal zone B and B1 cell-specific protein	9.5e-08	2.3e-06	Lymphoid tissues.	↑	Promotes IgM assembly and secretion.
DTNB	Dystrobrevin, beta	2.0e-05	3.6e-04	Muscle, kidney, pancreas, brain.	↑	None identified.

LAX1	Lymphocyte transmembrane adaptor 1	5.1e-06	5.1e-05	T and B cells.	↑	Negatively regulates T and B cell antigen receptor-mediated signaling.
IGLV3-25	Immunoglobulin lambda variable 3-25	2.5e-06	3.9e-05	B cells.	↑	Immunoglobulin light chains.<br>.
IGKV4-1	Immunoglobulin kappa variable 4-1	4.7e-06	3.2e-05	B cells.	↑	Light chain of the B cell receptor; secreted by B cells.
ITM2A	Integral membrane protein 2A	2.7e-05	3.5e-04	T cells, various tissues.	↑	None identified
PIM2	Pim-2 oncogene	3.8e-06	9.4e-05	Various tissues; neoplasms.	↑	*Oncogene.*
MXI1	MAX interactor 1, dimerization protein	7.8e-05	2.0e-03		↓	*Putative tumor suppressor which negatively regulates MYC function.*
IGHM	Immunoglobulin heavy constant mu	1.7e-05	1.3e-04	B Cells.	↑	B cell antigen receptor complex; Igs.
INPPL1	Inositol polyphosphate phosphatase-like protein 1	7.9e-05	8.2e-04	Macrophages, various tissues.	↓	Negative regulator of the immune system.

[1]The Chi-squared probabilities were calculated by ANOVA for all 24 logistic regression gene models. [2]The arrows indicate the relative expression levels in the long survivors group when compared to the levels in the early death group.
