# Supplementary material for: Using logistic regression to improve the prognostic value of microarray gene expression data sets: application to early-stage squamous cell carcinoma of the lung and triple negative breast carcinoma
Source: BMC Med Genomics. 2014 Jun 10;7:33. doi: 10.1186/1755-8794-7-33 (PMC4110620; doi:10.1186/1755-8794-7-33)
Supplement: Additional file 3: Table S3 — The entire list of consensus prognostic genes identified by the revolving sliding window approach. [file 1755-8794-7-33-S3.pdf]

Table S3. The entire list of consensus prognostic genes identified by the revolving sliding window approach.

| NUMBER | GENE NAME | ACCURACY COUNT |
|---|---|---|
| 1 | LSP1 | 2 |
| 2 | ACSBG2 | 3 |
| 3 | POSTN | 3 |
| 4 | MMP1 | 3 |
| 5 | HOXC8 | 3 |
| 6 | COL3A1 | 4 |
| 7 | PDZRN3 | 5 |
| 8 | WNT2 | 5 |
| 9 | CEND1 | 5 |
| 10 | FCRL2 | 6 |
| 11 | SLC19A1 | 6 |
| 12 | PDGFRL | 6 |
| 13 | KCTD3 | 6 |
| 14 | SNUPN | 7 |
| 15 | PNOC | 7 |
| 16 | ISG20 | 8 |
| 17 | C1S | 9 |
| 18 | NEUROG1 | 10 |
| 19 | YBX2 | 11 |
| 20 | CCL2 | 11 |
| 21 | CSTA | 12 |
| 22 | ITGB7 | 12 |
| 23 | PRDM1 | 12 |
| 24 | FAM46C | 12 |
| 25 | CCDC88C | 13 |
| 26 | FMO3 | 14 |
| 27 | CD83 | 14 |
| 28 | METTL2B | 14 |
| 29 | IGL@ | 14 |
| 30 | KIAA0125 | 16 |
| 31 | C2CD2L | 16 |
| 32 | IGJ | 17 |
| 33 | RHOH | 18 |
| 34 | GUSBP11 | 19 |
| 35 | DCN | 19 |
| 36 | INPPL1 | 22 |
| 37 | IGHM | 23 |
| 38 | MXI1 | 23 |
| 39 | PIM2 | 24 |
| 40 | ITM2A | 25 |
| 41 | IGKV4-1 | 25 |
| 42 | IGLV3-25 | 26 |
| 43 | LAX1 | 26 |
| 44 | DTNB | 28 |

Table S3. The entire list of consensus prognostic genes identified by the revolving sliding window approach.

| | | |
|---|---|---|
| 45 | MZB1 | 29 |
| 46 | IGLL3P | 30 |
| 47 | IGKC | 31 |
| 48 | POU2AF1 | 33 |
| 49 | IGHG1 | 34 |
| 50 | TNFRSF17 | 34 |
| 51 | IGHD | 34 |
| 52 | VPREB3 | 34 |
| 53 | CPA3 | 36 |
| 54 | GM2A | 38 |
| 55 | IGLJ3 | 40 |
| 56 | IGLV1-40 | 41 |
| 57 | IGLV3-19 | 42 |
| 58 | CD27 | 47 |
| 59 | CD79A | 48 |
