# Supplementary material for: Using logistic regression to improve the prognostic value of microarray gene expression data sets: application to early-stage squamous cell carcinoma of the lung and triple negative breast carcinoma
Source: BMC Med Genomics. 2014 Jun 10;7:33. doi: 10.1186/1755-8794-7-33 (PMC4110620; doi:10.1186/1755-8794-7-33)
Supplement: Additional file 4: Table S4 — Correlation coefficients of CD79A versus the remaining 23 prognostic genes. [file 1755-8794-7-33-S4.pdf]

Table S4. Correlation coefficients of CD79A versus the remaining 23 prognostic genes.

GENE NAME	CORRELATION WITH CD79A
IGLL3P	0.88
LAX1	0.87
IGLJ3	0.87
IGHM	0.85
MZB1	0.85
IGKC	0.85
PIM2	0.83
IGLV3-25	0.82
IGLV1-40	0.82
POU2AF1	0.80
CD27	0.78
TNFRSF17	0.78
IGHG1	0.77
IGHD	0.77
IGLV3-19	0.77
IGKV4-1	0.75
DTNB	0.66
ITM2A	0.47
CPA3	0.46
VPREB3	0.34
GM2A	0.25
INPPL1	-0.059
MXI1	-0.26
