# Supplementary material for: Using logistic regression to improve the prognostic value of microarray gene expression data sets: application to early-stage squamous cell carcinoma of the lung and triple negative breast carcinoma
Source: BMC Med Genomics. 2014 Jun 10;7:33. doi: 10.1186/1755-8794-7-33 (PMC4110620; doi:10.1186/1755-8794-7-33)
Supplement: Additional file 5: Table S5 — Forty genes identified by KM analysis of stage I and II cases from the GDS2373 data set. [file 1755-8794-7-33-S5.pdf]

Table S5. Forty genes identified by KM analysis of stage I and II cases from the GDS2373 data set.

IGLJ3
CYP20A1
IGKC
MPI
IGHD
GM2A
TNFRSF25
CDC25C
DTNB
SYNE1
GABRR1
EVL
IGKV4-1
TNFRSF17
RUNX3
ALDH7A1
CD79A
CHKA
POU2AF1
CD27
TSPO
MZB1
PPAP2A
INPPL1
KRT7
CPA3
CCDC87
TBC1D16
UPP1
OXCT1
GLRA2
LGALS3BP
HLA-DOB
HSD17B6
ELAC1
PFKP
LRBA
IGLL3P
AKTIP
CCDC88C
