# Supplementary material for: Using logistic regression to improve the prognostic value of microarray gene expression data sets: application to early-stage squamous cell carcinoma of the lung and triple negative breast carcinoma
Source: BMC Med Genomics. 2014 Jun 10;7:33. doi: 10.1186/1755-8794-7-33 (PMC4110620; doi:10.1186/1755-8794-7-33)
Supplement: Additional file 6: Table S6 — A list of 63 Affymetrix HGU133A CEL files used for the TNB study. [file 1755-8794-7-33-S6.pdf]

Table S6. A list of 63 Affymetrix HGU133A CEL files used for the TNB study.

GSM79306.CEL	GSM282427.CEL
GSM79336.CEL	GSM282457.CEL
GSM120702.CEL	GSM282474.CEL
GSM177909.CEL	GSM282493.CEL
GSM36949.CEL	GSM282528.CEL
GSM79329.CEL	GSM177897.CEL
GSM120668.CEL	GSM177913.CEL
GSM65839.CEL	GSM178021.CEL
GSM36927.CEL	GSM178023.CEL
GSM282454.CEL	GSM178065.CEL
GSM282551.CEL	GSM178078.CEL
GSM65878.CEL	GSM177980.CEL
GSM36797.CEL	GSM177985.CEL
GSM120687.CEL	GSM178016.CEL
GSM178009.CEL	GSM178034.CEL
GSM177993.CEL	
GSM120655.CEL	
GSM282569.CEL	
GSM36952.CEL	
GSM177999.CEL	
GSM178079.CEL	
GSM36835.CEL	
GSM177935.CEL	
GSM282398.CEL	
GSM177899.CEL	
GSM37002.CEL	
GSM36905.CEL	
GSM282413.CEL	
GSM177956.CEL	
GSM37050.CEL	
GSM37051.CEL	
GSM782528.CEL	
GSM79115.CEL	
GSM79145.CEL	
GSM79165.CEL	
GSM79280.CEL	
GSM79356.CEL	
GSM65842.CEL	
GSM65845.CEL	
GSM36855.CEL	
GSM36876.CEL	
GSM36961.CEL	
GSM36977.CEL	
GSM120649.CEL	
GSM120658.CEL	
GSM120683.CEL	
GSM120684.CEL	
GSM120696.CEL	
