# Supplementary material for: Using logistic regression to improve the prognostic value of microarray gene expression data sets: application to early-stage squamous cell carcinoma of the lung and triple negative breast carcinoma
Source: BMC Med Genomics. 2014 Jun 10;7:33. doi: 10.1186/1755-8794-7-33 (PMC4110620; doi:10.1186/1755-8794-7-33)
Supplement: Additional file 7: Table S7 — TNB logistic regression analysis: AUC of 203 immune related genes. [file 1755-8794-7-33-S7.pdf]

Table S7. TNB logistic regression analysis: AUC, 203 immune-related genes.

NUMBER	IMMUNE GENE	AUC
1	AIRE	0.5225
2	ALK	0.675
3	BANK1	0.8575
4	BCAP29	0.5625
5	BCAP31	0.7
6	BCL10	0.495
7	BCL11A	0.4575
8	BCL11B	0.5975
9	BCL2	0.3775
10	BCL3	0.56
11	BCL6	0.505
12	BCL7A	0.6725
13	BCL7B	0.5225
14	BCL7C	0.53
15	BCL9	0.6525
16	BLK	0.4775
17	BLNK	0.8
18	BTG1	0.6725
19	BTG4	0.6375
20	CD27	0.76
21	CD79A	0.7275
22	CD79B	0.7325
23	CPA3	0.45
24	CTAGE1	0.72
25	CTLA4	0.53
26	DLEU1	0.6875
27	DLEU2	0.545
28	DTNB	0.6825
29	EBF2	0.5525
30	EPAG	0.59
31	FRAT1	0.5225
32	FRAT2	0.505
33	GM2A	0.6575
34	GZMA	0.7325
35	GZMB	0.6875
36	GZMM	0.6475
37	HELLS	0.5475
38	HIVEP1	0.5675
39	HIVEP2	0.5775
40	HIVEP3	0.575
41	ICOS	0.735
42	ICOSLG	0.55
43	IGBP1	0.5775
44	IGHD	0.7575
45	IGHG1	0.605
46	IGHM	0.6925
47	IGHMBP2	0.705
48	IGHV3-48	0.76
49	IGHV5-78	0.69
50	IGJ	0.6775
51	IGK@	0.74
52	IGKC	0.735
53	IGLC1	0.685
54	IGLJ3	0.725
55	IGLL1	0.585
56	IGLL3P	0.6675
57	IGLL5	0.765
58	IGLV1-44	0.7825
59	IGLV6-57	0.72
60	IGSF1	0.6275
61	IGSF3	0.63
62	IGSF6	0.655
63	IGSF9B	0.5375
64	IKBKAP	0.495
65	IKBKB	0.5275
66	IKBKE	0.53
67	IKBKG	0.6025
68	IL12A	0.57
69	IL12B	0.7025
70	INPPL1	0.615
71	IRGC	0.5875
72	IRGQ	0.62
73	ISLR	0.64
74	ITGAE	0.5475
75	ITGAL	0.7875
76	ITK	0.675
77	ITM2A	0.785
78	KIR2DL2	0.6575
79	KIR2DL3	0.66
80	KIR2DL4	0.625
81	KIR2DS1	0.685
82	KIR2DS3	0.7625
83	KIR2DS5	0.58
84	KIR3DL1	0.6875
85	KIR3DL3	0.635
86	KIR3DX1	0.7025
87	LAG3	0.5675
88	LAIR1	0.61
89	LAIR2	0.545
90	LAX1	0.75
91	LCK	0.745
92	LCP1	0.7425
93	LCP2	0.755
94	LEF1	0.5275
95	LILRA1	0.5775
96	LILRA2	0.4575
97	LILRA3	0.6425

Table S7. TNB logistic regression analysis: 203 immune related genes with an AUC of > 0.65.

98	LILRA4	0.755
99	LILRA5	0.55
100	LILRA6	0.565
101	LILRB1	0.67
102	LILRB2	0.65
103	LILRB4	0.725
104	LILRB5	0.55
105	LILRP2	0.66
106	LRIG1	0.5475
107	LRIG2	0.595
108	LRIT1	0.53
109	LRMP	0.6325
110	LSP1	0.665
111	LTA	0.6225
112	LTB	0.8075
113	LTBR	0.5875
114	LY6D	0.54
115	LY6E	0.6075
116	LY6G5C	0.5775
117	LY6G6C	0.75
118	LY6G6D	0.59
119	LY6G6E	0.5575
120	LY6H	0.5225
121	LY75	0.565
122	LY86	0.755
123	LY9	0.695
124	LY96	0.6575
125	LYL1	0.7275
126	LYVE1	0.7775
127	MAL	0.505
128	MALL	0.625
129	MALT1	0.5825
130	MILR1	0.715
131	MLL	0.5525
132	MLL2	0.725
133	MLL4	0.5625
134	MLLT1	0.695
135	MLLT10	0.635
136	MLLT10P1	0.585
137	MLLT11	0.59
138	MLLT3	0.645
139	MLLT4	0.69
140	MXI1	0.52
141	MZB1	0.73
142	NFAT5	0.54
143	NFATC1	0.535
144	NFATC2IP	0.56
145	NFATC3	0.6375
146	NFATC4	0.68
147	NFKB1	0.5225
148	NFKB2	0.7875
149	NFKBIA	0.6175
150	NFKBIB	0.6275
151	NFKBIE	0.705
152	NFKBIL1	0.5475
153	PBX1	0.5025
154	PBX2	0.5475
155	PBX3	0.4625
156	PBXIP1	0.5225
157	PIBF1	0.6325
158	PIGR	0.5725
159	PILRA	0.6525
160	PILRB	0.5875
161	PIM2	0.765
162	POU2AF1	0.695
163	PTCRA	0.5225
164	PTPN22	0.72
165	PVRIG	0.68
166	RBPJ	0.5475
167	RBPJL	0.65
168	SEMA3A	0.535
169	SEMA3B	0.5825
170	SEMA3C	0.56
171	SEMA3D	0.6025
172	SEMA3E	0.555
173	SEMA3F	0.5425
174	SEMA3G	0.56
175	SEMA4A	0.5325
176	SEMA4C	0.695
177	SEMA4D	0.675
178	SEMA4F	0.67
179	SEMA4G	0.6425
180	SIGIRR	0.4875
181	SLAMF1	0.6725
182	SWAP70	0.5525
183	TAL1	0.6425
184	TAX1BP1	0.5825
185	TCF7	0.505
186	TCF7L1	0.6625
187	TCF7L2	0.5825
188	TCIRG1	0.515
189	TCL1A	0.745
190	TCL1B	0.525
191	TCL6	0.4925
192	TCTA	0.5075
193	TIAM1	0.6225
194	TIAM2	0.69
195	TIE1	0.4925

Table S7. TNB logistic regression analysis: 203 immune related genes with an AUC of > 0.65.

196	TLX1	0.6025
197	TLX2	0.77
198	TLX3	0.665
199	TNFRSF17	0.725
200	VPREB1	0.6975
201	VPREB3	0.7475
202	VSIG10	0.5275
203	VSIG4	0.56
