# Supplementary material for: Using logistic regression to improve the prognostic value of microarray gene expression data sets: application to early-stage squamous cell carcinoma of the lung and triple negative breast carcinoma
Source: BMC Med Genomics. 2014 Jun 10;7:33. doi: 10.1186/1755-8794-7-33 (PMC4110620; doi:10.1186/1755-8794-7-33)
Supplement: Additional file 8: Table S8 — TNB logistic regression analysis: 3 apparently non-immune related genes that have an AUC > 0.86, the best AUC score obtained with an immune gene. [file 1755-8794-7-33-S8.pdf]

Table S8. TNB logistic regression analysis: 3 apparently non-immune related genes that have an AUC > 0.8, the best AUC score obtained with an immune gene.

	Gene	Description	AUC
204960_at	PTPRCAP	protein tyrosine phosphatase, receptor type, C-associated protein	0.8725
214269_at	MFSD7	major facilitator superfamily domain containing 7	0.8625
220345_at	LRRTM4	leucine rich repeat transmembrane neuronal 4	0.885
