# Supplementary material for: Using logistic regression to improve the prognostic value of microarray gene expression data sets: application to early-stage squamous cell carcinoma of the lung and triple negative breast carcinoma
Source: BMC Med Genomics. 2014 Jun 10;7:33. doi: 10.1186/1755-8794-7-33 (PMC4110620; doi:10.1186/1755-8794-7-33)
Supplement: Additional file 9: Table S9 — TNB logistic regression analysis: immune genes that are closely correlated with gene IGLV1-44 in the initial TNB data analysis; also given are the AUC values for each of the genes. [file 1755-8794-7-33-S9.pdf]

Table S9. TNB logistic regression analysis: immune genes that are closely correlated with gene IGLV1-44 in the initial TNB data analysis; also given are the AUC values for each of the genes.

GENE	AUC	CORRELATION WITH IGLV1-44
SEL1L	0.61	0.71
DNAJB9	0.63	0.71
SKAP2	0.81	0.66
KIAA0125	0.68	0.69
CLN3	0.72	0.67
IGHM	0.69	0.88
GM2A	0.66	0.67
IGHG1	0.61	0.76
IGLJ3	0.73	0.89
GUSBP11	0.63	0.79
IGKC	0.74	0.84
IGLC1	0.69	0.71
IGLV1-44	0.78	1.00
UBE2J1	0.70	0.65
FKBP11	0.61	0.69
GADD45A	0.72	0.65
PIM2	0.77	0.71
CD79A	0.73	0.79
POU2AF1	0.70	0.81
CD27	0.76	0.76
TNFRSF17	0.73	0.86
LAX1	0.75	0.76
CYTIP	0.74	0.67
IGK@	0.74	0.87
IGJ	0.68	0.77
IGHD	0.76	0.87
IGLL3P	0.67	0.77
IGLL5	0.77	0.93
IGHV3-48	0.76	0.85
SPCS1	0.52	0.67
SLAMF7	0.75	0.70
FAM46C	0.64	0.71
TXNDC15	0.67	0.73
MZB1	0.73	0.84
DEF6	0.77	0.65
